# Identification of Children of Mentally Ill Patients and Provision of Support According to the Norwegian Health Legislation: A 11-Year Review

**DOI:** 10.3389/fpsyt.2021.815526

**Published:** 2022-01-14

**Authors:** Charlotte Reedtz, Eva Jensaas, Trine Storjord, Kjersti Bergum Kristensen, Camilla Lauritzen

**Affiliations:** ^1^Faculty of Health Science, Regional Centre for Child and Adolescent Mental Health and Child Welfare (RKBU North), Arctic University of Norway, Tromsø, Norway; ^2^University Hospital of North Norway, Tromsø, Norway

**Keywords:** change of clinical practice, children of mentally ill parents, provision of support, prevention of socio-emotional problems, identification of risk status

## Abstract

**Background:**

According to amended legislation implemented in Norway in 2010, personnel in healthcare services for adults are obligated to identify patients' minor children and to assess the family situation. Health personnel is also obligated to contribute to adequate support to families affected by parental mental illness or substance use disorders. The intention behind the amendment was to support and protect children of mentally ill parents, as they are at risk of developing problems of their own. The aim of the present study was to evaluate health personnel's practice during the years 2010-2020, more specifically; (a) to what extent children of patients with mental illness and substance use disorders are registered in patient records, and (b) to what extent activities relating to the assessment and support of patients' minor children are documented in patient records.

**Method:**

The participants in the study are patients admitted to Division for Mental Health and Substance Use at the University Hospital of North Norway in the years 2010–2020. The data was drawn from patient records during October 2021.

**Results:**

The registration of patients' minor children is considerably strengthened since the introduction of the new Norwegian Health Personnel Act in 2010, and estimates show that 56% of patients' minor children are identified. However, only 31% of cases where patients have identified minor children this result in health personnel performing activities to support the children.

**Discussion:**

Based on the rising proportion of identified minor children throughout the 10-year period, it seems evident that the dissemination efforts have contributed to the development of some new skills among health personnel. However, compared with the national estimation that 35% of mentally ill and substance abusing patients have minor children, a large proportion of children remains unidentified. After identification, there seem to still be a long way to go before minor children are systematically offered support. Different solutions to strengthen the implementation of new skills in clinical practice, to ensure the identification of minor children and provision of necessary support for them is discussed.

## Transgenerational Transmission of Mental Disorders

The transgenerational transmission of mental disorders is a significant cause of mental illness, and children of parents with mental illness or substance use disorders (COPMI) are at risk of developing mental illness themselves (1). Elevated risk for COPMI has been demonstrated across the diagnostic spectrum and is both diagnosis-specific and general (2). In addition, parents' symptomatology also have an impact on their social functioning and may in turn shape the way parents interact with their children. Impairment of parenting skills, reduced quality of care parents provides and problems in the parent-child interactions, is often a result of psychopathology in parents (3, 4). Furthermore, such impairments may in turn lead to insensitivity, hostility directed at the child, rejection and neglect (3), with possible subsequent insecure attachment (5, 6), emotional dysregulation, negative emotionality and pathological coping strategies as well as psychopathology in childhood, adolescence and adulthood (1, 3). As a consequence of hereditary, social and parent-child interaction factors, COPMI are very likely to constitute the next generation of mentally ill persons and parents (7).

Many children live in families with parental mental health problems and one in five has a parent with mental illness (8). In Norway, it has been estimated that 450,000 minor children (41.5 % of all children) have parents with mental illness or alcohol use disorder (9). The National Institute of Public Health (NIPH) has calculated this based on prevalence studies of the number of adults who qualify for a psychiatric diagnosis or alcohol dependency in 1 year. The numbers are adjusted for the fact that people with diagnosable mental illnesses have children to a smaller degree compared to healthy people, and that they often find partners with diagnosable mental illnesses. Since the estimate represent a cross section of the data throughout a year, consequently the number of children with parents with diagnosable mental illnesses throughout their childhood is even higher. Other researchers (10) have also estimated that one third of the patients at Norwegian hospitals have care responsibilities for minor children. Even though there is a solid evidence base for the many risk factors related to the transgenerational transmission of mental illnesses, research shows that it has been very difficult for professionals working with mentally ill patients and substance use problems to identify and support their children (2, 11, 12). In Norway, these children have not traditionally been registered in their parents' records and hence have not been identified. Without routines to assess whether patients have children, it is impossible to safeguard children who are affected by parental mental illness and related family problems. For these reasons, Norwegian authorities made legislative amendments in 2010 to safeguard minor children (0–18 years) of patients with mental illnesses and substance use disorders by adding new paragraphs to the Norwegian Health Personnel Act (13). Health personnel have since 2010 been required to provide minor children with information and necessarily follow up related to parental mental illness and substance use. The new regulations require all health professionals to; (1) register dependent children in the patient's record, (2) inform the parent about children's need for information and support, (3) assist in providing the children in the family with information, (4) provide the children with information about the opportunities to visit parents at the hospital, (5) assess children's and the family's needs, and (6) obtain parents' consent to cooperate with other services in establishing necessary support (14).

## Implementation of Practice Changes

Implementation is defined as targeted effort to carry out plans, decisions or interventions in a municipality, organization or general practice (15). This definition implies that implementation processes are targeted, managed and described in great enough details for independent observers to be able to observe the process and evaluate it. A model for degrees of implementation was developed by Fixsen et al. (15). The model categorizes goals and results of an implementation process as either paper implementation, process implementation or skills implementation. In paper implementation, decisions about innovations are rooted in formal resolutions. In process implementation, procedures and systems are changed to make it possible to materialize the innovations, and relevant participants are provided with necessary training. In skills implementation, relevant participants are conducting the innovation in such a way that new skills are manifested in clinical practice and can be measured. The legislation related to COPMI represent paper implementation according to Fixsen et al.'s model, as the planned innovation was rooted in new paragraphs in existing legislation and regulations related to these. The legislative authorities also described how the new laws and regulations should be operationalized, thus representing the next level in the implementation model, process implementation.

Changing human behavior is however challenging (16). This is also true for changing health personnel's practice related to patient's minor children (11, 17). Implementation science focuses on studying methods for promoting the uptake of consolidated research findings into routine healthcare practice and health policy, and many researchers have studied which factors have an impact on the implementation of new practices (16, 18, 19). A recent scoping review conducted by Fakha et al. (18), identified an interplay of 25 main factors that acted as barriers and facilitators during the implementation of diverse health care innovations (18). There is a wide range of interrelated factors existing at multiple levels that determine the success of the implementation of innovations (18), which explains why changing clinical practice is challenging, time consuming and needs to be monitored over time.

In a previous study at the University Hospital in North Norway (UNN), which is the largest hospital in the region, results showed that only 4–7% of patients were registered with minor children, even though 35.3% of patients were estimated to have minor children (20). Given the speed of implementation in year 2015, it was calculated that it would take ~19 years before patients' children were registered, and hence identified the way they should according to the amended legislation from 2010. There may be many reasons to why health personnel do not register or identify patients' children in patient records. First, they may have low professional awareness related to the fact that many patients are caring for minor children, and that these children are at risk of developing social and/or mental health problems themselves (21). Second, health personnel who work with adult patients may feel insecure in discussions about childcare and in including patients' children in the treatment, because they are not trained or educated to do this (12). Third, the financial structure of health services in Norway is based on client contacts and as patients' children are not clients, contact with COPMI does not result in any financial support or refund. Fourth, it may be unclear whose responsibility it is to register patients' children in the journal. Fifth, time constraints in clinical work at hospitals may result in health personnel not prioritizing assessment of whether the patient has children or the needs of these children (22). A recent study showed that the rate of registering patients' minor children was higher in university hospitals compared to smaller hospitals in the country, and that Norwegian hospitals had implemented change in clinical practice related to COPMI at a medium level (13).

## Specific Actions to Support Implementation of the Amended Legislation in North Norway

A crucial instrument to change the clinical practice related to patients' minor children, was to make it mandatory for all hospitals in Norway to appoint child responsible personnel (CRP) in wards, clinics and institutions. The intention was that CRP should be responsible for promoting and coordinating support for patients' minor children (23). The University Hospital in North Norway (UNN) also chose to establish a new function named CRP-coordinator in each clinic and these served as managers of all CRP in their clinic. Furthermore, The Northern Norway Regional Health Authority made guidelines to describe the mandated clinical practice to identify and support COPMI, and these were effective from 2012. The guidelines described which information about COPMI should be registered, who should register, how to document the information in the electronic patient records (EPR), as well as where in the EPR this information belonged. The EPR utilized by the hospitals in North Norway is called DIPS, which is the largest supplier of eHealth systems to Norwegian hospitals. DIPS provides a software package for EPR, which in turn provide health care workers with an integrated and unified electronic presentation of all important and relevant clinical information about patients, including patients' minor children. In DIPS patients' minor children should be registered at the front page in the EPR, among central patient administrative information.

In addition to the provision of specific guidelines and procedures related to the process implementation of the innovation, The Northern Norway Regional Health Authority provided health personnel with opportunities to participate in training programs related to service provision for COPMI. From 2013 they financed even larger parts of the implementation process, and a considerable sum were used to train health personnel and to support implementation activities. In 2014, UNN was also provided with a 50% position as CRP coordinator managing all CRP and COPMI related activities in the hospital, in order to support implementation activities related to the innovation. In 2016 a National Professional Procedure for patients with minor children was implemented and approved by the National Health Library. This procedure and the guidelines from the regional health authority coincided and put even greater pressure to change clinical practice accordingly. Furthermore, from 2017, UNN decided to change the terms for CRP-coordinators in each clinic and chose to pay health personnel in 20% positions to support the implementation process. In sum, The Northern Norway Regional Health Authority have taken control over a variety of implementation activities, and it seems safe to say that the legislative changes related to COPMI in UNN has been followed by implementation support at both the local, regional and national level. However, until now, it remains unclear whether the implementation of the innovation represents what could be characterized as changed clinical practice through acquirement of new skills related to COPMI.

## The Practice Changes to be Monitored Over Time

In this particular study, the practice changes to be monitored over time is linked to the amended legislation (the new paragraphs in the Norwegian Health Personnel Act from 2010), where the registration of COPMI was the core intention, as well as documenting activities related to provision of support for patients' minor children. All COPMI activities should be documented in the EPR by a COPMI report. The COPMI report should include information about identification and assessment of the child, conversations about the child and family with the patient, conversations with the child and family, consent or no consent to cooperate with other services, evaluations of the situation of the child, as well as further follow-up. The COPMI reports have changed throughout the ten-year period from several separate documents to five documents which can be utilized in the EPR in 2020. As of now, the main COPMI report is designed as a form which could be continued with new entries as new activities are performed in clinical practice. The intention with this report is to provide an easily accessible overview of relevant information regarding the patient's minor children and how they have been informed and supported. According to the mandatory guidelines, all patients with mental illnesses or substance use disorders who are registered with minor children should have at least one COPMI related documents in EPR and this is a COPMI report.

## Aims of the Current Study

The present study is part of a longitudinal COPMI project at the Arctic University of Norway, in which the goal was to support the implementation of new routines arising from legislative amendments, as well as to evaluate the process of change (20). The aim of the present study was to evaluate (a) to what extent health personnel registered children of patients with mental illness and substance use in electronic patient records (EPR) during the years 2010–2020, and (b) to what extent activities relating to the assessment and support of COPMI are documented in EPR according to the mandatory guidelines.

## Methods

### Participants

**Figure 1:**
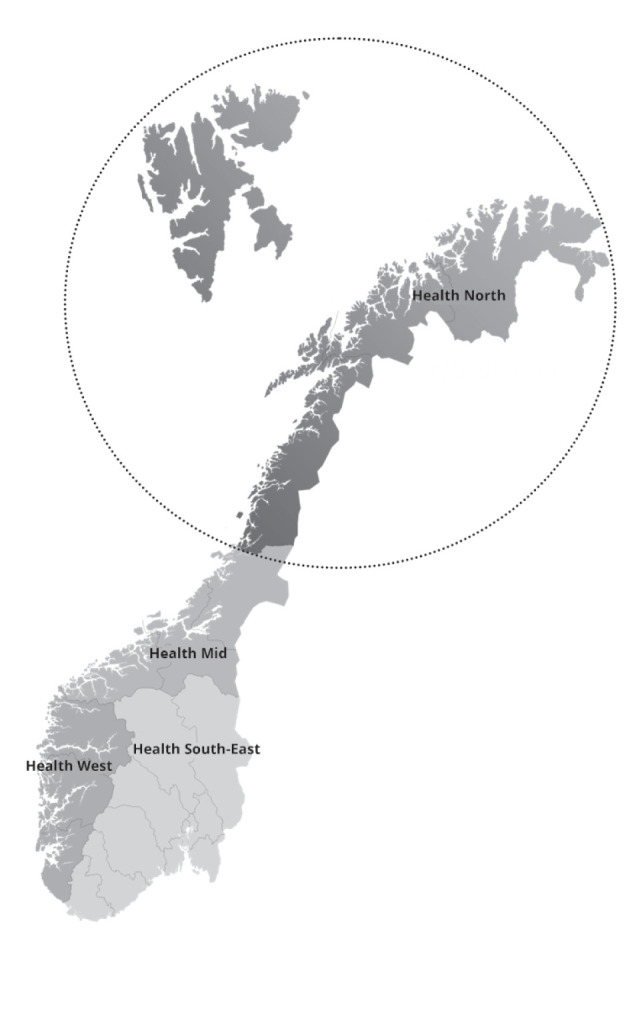
Norwegian Regional Health Authorities. The Northern Norway Regional Health Authority in circle.

Participants in this study were all patients in the Division for Mental Health and Substance Use disorders in the largest hospital in North Norway. The University Hospital in North Norway (UNN) is responsible for the delivery of mental health care services at the specialist level in the two most northern counties in Norway (see Figure 1). Northern Norway is an area with large geographical spread, covering an area two times larger than Denmark. UNN is one of four public health undertakings in the region, all part of The Northern Norway Regional Health Authority.

### Design and Procedure

This study is a retrospective registry study based on anonymized data from EPR. Data from all patients were drawn from DIPS EPR in October 2021 and consisted of:

- Information regarding the patients' children, registered in the administrative front page of the EPR.
- Information regarding COPMI related activities, registered in COPMI reports in the EPR.

### Recruitment

UNN's participation in this quality assurance study is rooted in a collaboration between The Arctic University of Norway - UiT and a project manager at UNN.

### Ethical Considerations

Quality assurance studies in UNN do not require patient consent. All analyzed data was anonymized. The project has been approved by the Data protection officer at UNN.

### Statistical Analyses

Statistical analyses were computed using SPSS (version 19) and Excel^®^ for Microsoft 365 MSO.

Based on data from Statistics Norway (SSB) we have estimated the probability of an adult person having children between the age of 0 and 18 years old. Using Bayes' formula (24) we calculated the probability of mentally ill adults in Norway having children. These calculations are based on statistical information from SSB and the Norwegian Institute of Public Health (NIPH). SSB provides data on the number of households in Norway, the portion of married, co-habitants and single adults, as well as to what degree people in these groups have children. In a study from NIPH (9) researchers estimated the relative risk of being mentally ill when you have children vs. being mentally ill in the general population. We included this information in our calculations and calculated the probability for the degree to which mentally ill patients in Norway have children. Our analyses showed that the chance that mentally ill adults in Norway have children is 33.5%. In addition to that the Norwegian Institute of Public Health has estimated that there is a 5.4% elevated risk to have mental illness when one is a parent, compared to the risk of this in the general adult population (9). We added this risk into our calculations and the result showed that there is a 35.3% chance to have children when adults are mentally ill (25).

Time series modeling in SPSS was used to predict how many years it would take to adhere to the amended legislation related to COPMI.

## Results

### Patients Registered With Minor Children in EPR

A total of 28,906 unique patients received mental health care at Division for Mental Health and Substance Use Disorders at UNN during the years 2010–2020, in outpatient and inpatient units/wards at several locations (see Table 1). The mean age of patients ranged from 39 (median 38) in 2010 to 38 (median 35) in 2020. The results show that the number of patients with registered children in the years 2010–2020 increased rapidly from the first year with new legislation and onward. Based on the estimated probability of 35.3% that mentally ill patients in Norway have children, results show that the number of patients with minor children vary from 2,204 to 2,524 during the period from 2010 to 2020. The percentage of registered COPMI, based on these estimates, have increased from 0.1% in 2010, to 27.8% in 2015 and to almost 56% in 2020.

**Table 1:** Total number of patients per year, estimated number of patients with minor children, actual number of patients with registered minor children, and actual number of patients with registered minor children and at least one COPMI report documenting mandated COPMI activities in the EPR.

	**2010**	**2011**	**2012**	**2013**	**2014**	**2015**	**2016**	**2017**	**2018**	**2019**	**2020**
Total number of patients^*^	6,244	6,563	6,695	6,579	6,612	6,950	6,952	7,080	7,150	6,981	6,507
Estimated number of patients with minor children	2,204	2,317	2,363	2,322	2,334	2,453	2,454	2,499	2,524	2,464	2,297
Number of patients with minor children^**^	2	51	181	280	484	682	844	977	1,088	1,188	1,286
Number of patients with minor children and at least on registered document related to COPMI^***^	20	230	181	204	189	244	279	268	343	369	401

* *Data from HN LIS available from The Northern Norway Regional Health Authority*.

** *Data collected by using DIPS Report 2531765*.

*** *Data collected by using DIPS Report 2531754*.

Many patients are however patients over a longer period of time than 1 year or have been admitted more than one time during the 11-year period. In such cases they are counted as unique patients every year they were admitted as a patient in the Division for Mental Health and Substance Use Disorders, and hence possibly more than one time. Subsequently, minor children of these patients may also be registered every year they were admitted. Table 2 presents patients registered with minor children for the first time per year and the actual number of minor children these patients were registered with. Of the 455 children that were registered in 2019, a total of 238 of them were younger than 6 years of age. Results show that a total of 3,476 unique minor children have been identified during the 10-year period.

**Table 2:** Total number of patients registered with minor children for the first time per year, and the total number of children they were registered with in the EPR.

	**2010**	**2011**	**2012**	**2013**	**2014**	**2015**	**2016**	**2017**	**2018**	**2019**	**2020**
Total number of patients registered with minor children first time^*^	4	54	125	128	285	295	318	309	329	316	263
Total number of minor children registered first time^*^	4	60	166	177	391	405	443	442	466	455	467

* *Data collected by using DIPS Report 2531765*.

Based on the speed of changes related to the identification of patients' minor children from 2010 and onward the results show that it will take a total of 18 years until all minor children are identified, and hence this could be a reality in year 2028.

### COPMI Related Documents in EPR for Patients With Registered Minor Children

**Figure 2:**
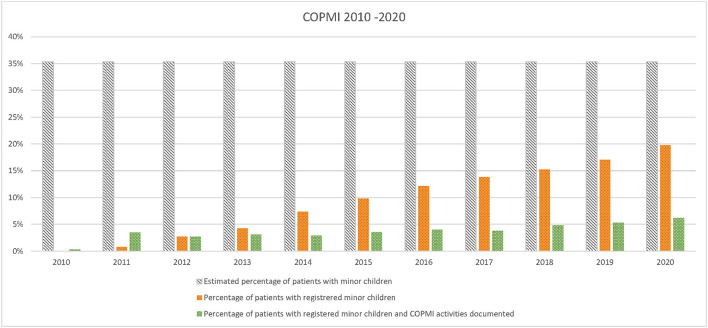
Estimated percentage of patients with minor children, actual percentages of patients with registered minor children and percentages of percentages of patients with registered and one document related to mandated COPMI activities in 2010–2020.

The results show that COPMI related documents are registered in the EPR of 35.5% of patients with registered minor children or lower, during the 11-year period (see Figure 2).

**Figure 3:**
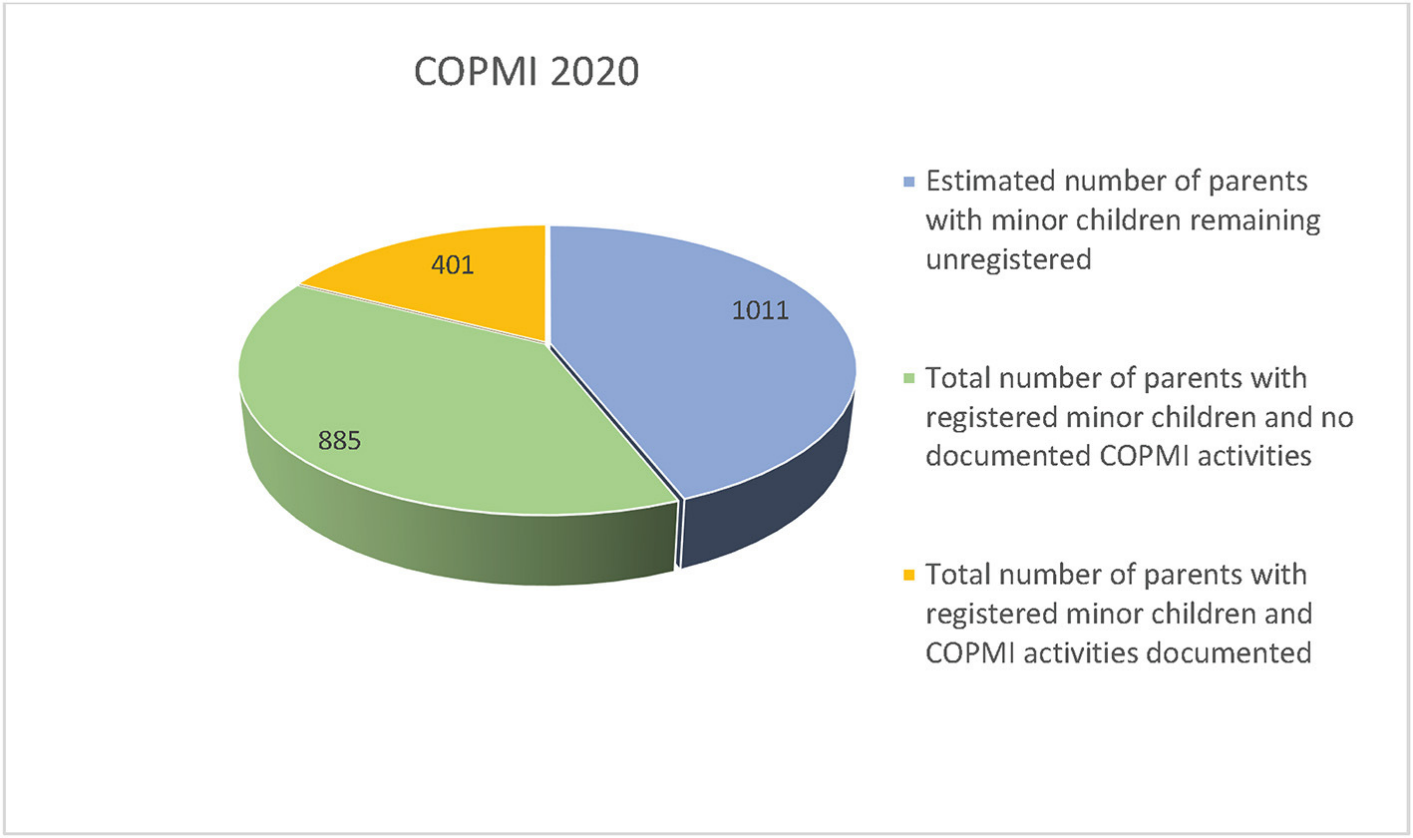
Total number of patients with minor children and patients with registered minor children and one document related to mandated COPMI activities in 2020.

Figure 2 shows that not every patient with registered minor children had at least one COPMI report in the EPR as mandated. In 2020 only 17.5% of patients estimated to have minor children had documented activities related to COPMI in EPR. Results show that during the last 3 years the number of patients with registered minor children and at least one document in EPR have stabilized at around 31% (31.5% in 2018, 31.1% in 2019 and 31.2% in 2020), indicating that more than two thirds of patients with registered minor children did not have mandated documents related to COPMI in the EPR. More specifically, the results show that in 2020 a total of 1,286 patients were registered with minor children (56%), whereas only 401 of these (31.2%) had registered documents related to COPMI in the EPR (see Figure 3). Figure 2 presents the estimated number of patients with unregistered minor children, actual number of patients with registered minor children and number of patients with registered minor children and at least one document related to mandated COPMI activities in 2020. The estimated number of patients with unregistered minor children in 2020 is 1,011.

Based on the speed of changes concerning documentation of COPMI related activities for patients with registered minor children the results show that it will take a total of 82 years from 2010 until this clinical practice is implemented, and hence this could be a reality in year 2092.

## Discussion

The first aim of the present study was to evaluate to what extent health personnel registered children of patients with mental illness and/or substance use disorders (COPMI) according to the Norwegian Health Personnel Act in electronic patient records (EPR) during the years 2010–2020. The result from this study shows that the identification of COPMI has improved considerably since the introduction of the new Norwegian Health Personnel Act in 2010. It is very positive that more children are identified, as this is a necessity to provide COPMI with the information and support they are entitled to. According to the straightforward procedures on how to register patients' children in the EPR, it seems that this is not time-consuming and is feasible for most health personnel.

Based on the rising proportion of identified COPMI throughout the 11-year period, it seems evident that the implementation steps and activities that have been utilized at UNN have contributed to the development of new skills among health personnel. The willingness to organize the implementation process, finance core activities and positions, as well as providing the workforce with training opportunities represents important steps to support development of COPMI related skills such as identifying patients' minor children.

However, compared with the national estimations that 35.3% of patients with a mental illness and/or substance use disorders have minor children, a large proportion of children still remains unidentified. To our knowledge, there are no reasons to believe that patients in the Division for Mental Health and Substance Use Disorders at UNN have children to a lower extent than the national estimates. Furthermore, there are no available data to support that the population of the two northernmost counties in Norway are parents to a lower extent than the rest of the country.

The actual age distribution among patients may also be relevant in this context, if the patients were too old to have children aged 0–18. However, our data shows that the mean age of patients in the Division for Mental Health and Substance Use disorders at UNN are in line with national reports on patient data where 70% of all patients in Norwegian mental health care is between 18 and 49 years. The national figures for age distribution among these patients are overlapping with our results, and hence most patients receiving mental health care services are in the age where the probability of having children aged 0–18 is very high. In addition, very few contacts or brief stays in the hospital per patient, could also explain the lack of registered minor children, because of the reduced time frame for doing this among health personnel. However, the mean amount of contacts per patient were five, and hence personnel had several chances to register patients‘ minor children.

Based on our results, it seems safe to conclude that not all health personnel have developed the skills to identify COPMI, and hence that the implementation has not moved beyond paper and procedural implementation for all. One implication of the result that an estimated number of 44% of patients with minor children are not registered with children, is that thousands of COPMI during the 11-year period are unidentified. These children may still be invisible to public services and are at even higher risk of developing social, emotional and mental health problem themselves, since they cannot be reached with effective support and/or interventions. It has taken 11 years to fill in about half of the gap between existing and identified COPMI. New steps are warranted to increase the number of identified children until all COPMI are identified according to the law.

Several changes in the implementation process may contribute to further improvement of the identification of COPMI. In previous studies researchers have pointed to changes in the software of the EPR as a source of strengthening the identification of COPMI (24). One suggested solution was that health personnel should not be able to make entries in EPR unless they had registered if the patient had minor children and had entered the names and birth dates of these children in the front administrative page of EPR. This would automatize identification skills among health personnel and result in full identification of COPMI. Another suggested solution was that patients' minor children were imported directly *via* the link between the EPR and the National Population Register, as for other patient variables such as id number. However, DIPS EPR is a complex software package, and changes like this have never been made.

Researchers have also suggested that the identification of patients' minor children and documenting COPMI related activities in EPR should be included as national quality indicators (13, 25). As such quality indicators also constitute the basis for the funding of the five regional health authorities in the country this could reinforce the adherence to the law and related guidelines for health personnel. The most recent recommendations included the establishment of national, regional and local implementation teams to strengthen the implementation support in all health care institutions (13). Skogøy et al. (13) have characterized the Norwegian process to implement legislation to protect COPMI as diverse and separate dissemination efforts, rather than a coherent implementation strategy (13). It is widely agreed that interventions to change professionals' practice need to be clearly specified (26). A coherent implementation strategy in this context should involve (a) defining the actions to be taken by health personnel, (b) an operationalization of the new practice, and (c) defining the mechanisms that are thought to result in change. In our view, the participating hospital has come a long way in terms of a and b but seem to lack a clear definition of the skills needed to fully implement the new practice as intended in the legislative amendments. According to Fixsen and colleagues' model of degrees of implementation, it is the skills level that represent the active mechanism for change (15). We believe there is still important work to do to define the skills needed in all health personnel in terms of identifying COPMI.

The second aim of the present study was to evaluate to what extent health personnel performed activities or interventions for minor children that was documented in the EPR according to the mandatory guidelines during the years 2010–2020. When a document concerning the patient's minor children is created in the EPR, it shows that a measurable activity related to provision of information and support to COPMI has been documented. The creation of such documents does not inform about the quality of activities, only the fact that it has been created. Therefore, such documents do not represent any form of quality assurance that the child is provided with the support they are entitled to. In order to evaluate that, one would have to enter each document and assess the quantity of the work documented in the reports. However, every patient in the Division for Mental Health and Substance Use Disorders at UNN with identified minor children should have at least one document concerning their minor children in their EPR. Lack of such documents, as the result in this study shows for the large majority of registered children, indicate that the mandatory guideline has not been followed, and hence that the implementation process has not reached the skills level for health personnel in this aspect either.

These finding are not unique, and in a study on the content of conversations with patients who are parents and conversations to support minor children (27), researchers explored data from EPR in 2010–2015. Results showed that very few patients registered with minor children received any type of documented parenting support, and that only a tiny fraction of registered children were included in conversations about their parents' mental health. Along with the results from the present study this clearly shows that the implementation and documentation of COPMI related activities lacks behind the identification of COPMI. After identification, there seem to still be a long way to go before COPMI are systematically offered support. This may be due to the lack of clearness around what this kind of activity should entail. Regardless of the reasons for the gap between registered minor children and the provision of support for these children, identifying COPMI and not offering support is ethically questionable in light of the existing knowledge about transgenerational transmission of mental disorders.

On the positive side, documentation in DIPS EPR is currently being developed so that procedural coding can be used to quantify different clinical activities at UNN. It is The Norwegian Directorate for Health and Social Affairs that are developing the codes, and DIPS implement them into EPR. Examples of such codes are family assessment of patients with minor children, conversations with patients about COPMI, conversations with COPMI, and collaboration with municipal services such as schools, day care centers, public health nurses, child welfare and protection services and so on. A total of 10 codes have been developed for COPMI so far and these activities can be coded in DIPS EPR.

However, even though these codes exist, health personnel are not mandated to use them yet. This means that health personnel may or may not code, and that whether they do or not have no consequences. A practice where health personnel utilize these codes for every patient with minor children will provide information about the quantity of all COPMI related activities in the future. Such practice will thus inform hospitals about to which degree they follow the law and provide children with necessary support and follow up. It is not a necessity that all patients with minor children have reports for each code, because some activities are based on consent from patients. This implies that only one or two coded activities may reflect a clinical practice in accordance with the law, if the patient did not consent for all possible activities. Furthermore, every patient and their children may not need all the same interventions. Many families struggling with mental health issues may have been identified in the health and social services in the communities where they live, and if this is the case many of them may receive support and interventions locally.

Coding registration of patients' minor children and activities related to COPMI in EPR may be experienced as an extra workload for health care personnel. It may however also motivate them. An example of how coding could be used as a motivational tool for COPMI, is how some diagnoses directly provides more funding for some wards. Serious malnutrition is one such diagnosis, and coding this diagnose provide wards with a specific amount of money per patient. To make such coding a mandatory part of the activity-based funding for hospitals seems like a reasonable step to prevent COPMI from being the next generation of mentally ill persons and parents. According to recently published principles and recommendations for working with children and parents living with parental mental illness (28), three take-home messages seem especially important for the adult mental health care services, even in Norway where legislative amendments have been made to protect minor children. These are:

- At intake, identify parenting status including pregnancy.
- Engage with clients in their parenting role and responsibilities.
- Engage with clients' children to identify, and respond to, their needs and/or initiate and coordinate agency referrals for children.

It seems that the participating hospital in this study has come a long way in terms of identification of parenting status, whereas engaging with parents about parenting issues and providing support for the children and families still needs to be developed.

## Data Availability Statement

The raw data supporting the conclusions of this article will be made available by the authors, without undue reservation.

## Ethics Statement

Ethical review and approval was not required for the study on human participants in accordance with the local legislation and institutional requirements. Written informed consent from the participants' legal guardian/next of kin was not required to participate in this study in accordance with the national legislation and the institutional requirements.

## Author Contributions

CR and EJ have contributed to the conceptualization of the study design. EJ, TS, and CR have retrieved and analysed the data. CR have put the paper in writing. CL, EJ, and KK contributed to commenting on and improving the manuscript text. All authors have approved of the final version of the paper.

## Conflict of Interest

The authors declare that the research was conducted in the absence of any commercial or financial relationships that could be construed as a potential conflict of interest.

## Publisher's Note

All claims expressed in this article are solely those of the authors and do not necessarily represent those of their affiliated organizations, or those of the publisher, the editors and the reviewers. Any product that may be evaluated in this article, or claim that may be made by its manufacturer, is not guaranteed or endorsed by the publisher.
